# Encouraging eye care workers to stay: the role of investment and management

**Published:** 2018-07-31

**Authors:** Suzanne Gilbert, Daksha Patel

**Affiliations:** 1 Senior Director: Innovation & Sight Programs, Seva Foundation, Berkeley, California USA.; 2 E-learning Director: International Centre for Eye Health, London School of Hygiene and Tropical Medicine, London, UK.

**Considering how expensive it is to train, recruit and employ eye health personnel, it makes sense to invest time and energy into creating a positive and productive working environment.**

**Figure:**
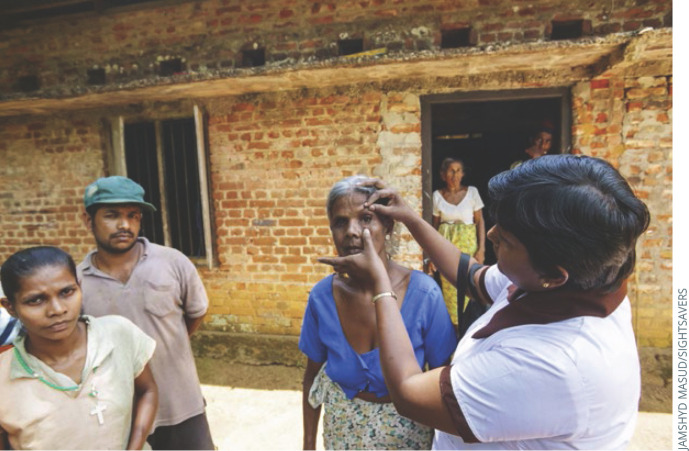
Delivering eye care to rural communities. SRI LANKA

Considerable effort (and often public money) is invested in the initial training, recruitment and placement of eye care professionals. It therefore makes good sense to support and motivate staff members to remain in their place of work – known as ‘retention’.

## Financial compensation and support

Good pay is an important factor in a person choosing to remain in post; particularly if there are attractive offers elsewhere. In the public sector, it may not be possible to offer substantial increases in direct wages; however, it may be possible to offer other financial incentives such as health insurance, pension schemes and/or allowances for child care, transportation and housing. Safe and secure accommodation, supplied with water and electricity, is a huge priority for staff members. Access to good schools – where practical – may also be a good incentive to stay. Financial incentives, although important, are not the only factors that influence retention. The conditions in which one works also have a powerful influence on job satisfaction, behaviour and, ultimately, retention.

## Good leadership and management

Managers and leaders set the tone in an organisation. Their values determine the kind of workplace it is for staff members; is it supportive and encouraging, or a ‘blame culture’, with an emphasis on fault finding?

Managers are most effective when they see themselves as providing:

- **Leadership**, e.g. by ensuring that staff members understand and share in the goals of the organisation.
- **Supportive supervision and feedback**, by means of regular review meetings that celebrate successes and review errors or challenges in a safe and supportive environment. Allowing staff members to get in touch with managers at any time, whether to discuss patient care or personal problems, is highly motivating.
- **An environment that supports effective service delivery**. Staff rosters and schedules must balance work with time off. Provide access to a car or other means e.g., a car or bike) and access to tools for communication and report writing (e.g., mobile phones and laptops).

**“We encourage our team members to take on new roles in teaching or outreach.”**

## Supportive human resource policies

Any organisation must have human resource policies in place that are responsive to staff members in terms of their personal, family and professional needs; for example, policies giving guidelines about annual and maternity leave. This can build staff members' connection to the institution.^1^ Equally important is having fair and transparent systems that highlight the duration of a posting (particularly to remote settings) and clear processes to address any difficulties or grievances in a supportive manner.

## Status and career advancement

Staff members are more likely to stay if they understand the potential career path open to them and know exactly what they must achieve in order to be promoted to a more responsible or demanding post with more benefits.

## Continuing professional education

Training enables health care professionals to set – and achieve – personal goals, retain their professional registration, and develop their skills and careers; all of which is motivating and improves their willingness to remain. Training existing staff members (for example, through online learning) can improve and extend the services offered at an eye clinic or hospital. This may be more cost-effective than recruiting new personnel.

## Recognition

There are many ways to express our appreciation of a person. These can be inexpensive, yet effective, such as regular ‘checking-in’ by the supervisor to see how personnel are doing or what they need, receiving a kind word from a manager/director to commend good work, or simply noting the birth or graduation of a family member. Awards or recognitions build the confidence of staff members and contribute toward the feeling of being valued and included.

## Teamwork

Working as part of a team is highly motivating. This requires first and foremost that the organisation genuinely values teamwork and ensures that everyone in the team clearly understands, accepts and values their own and each other's roles – whether clinical or non-clinical. Involve staff members in planning and identify what each member is willing to take responsibility. This ensures ownership – and pride! – when the team succeeds.

## Hospital infrastructure and community relations

The good reputation of an organisation within the community, smooth patient registration systems, functioning equipment, reasonable wait times, and pride in providing quality care all are big motivators for staff and patients. Demonstrate, and encourage staff members to develop, a relationship of mutual respect, trust and acceptance with the local community.
